# Development of a triplex crystal digital PCR for the detection of PRCoV, PRRSV, and SIV

**DOI:** 10.3389/fvets.2025.1562444

**Published:** 2025-03-31

**Authors:** Yuwen Shi, Kaichuang Shi, Yan Ma, Yanwen Yin, Feng Long, Shuping Feng, Meilan Mo, Jiakang He, Zuzhang Wei

**Affiliations:** ^1^Guangxi Key Laboratory of Animal Breeding, Disease Control and Prevention, College of Animal Science and Technology, Guangxi University, Nanning, China; ^2^Nanning Kedi Biotechnology Co., Ltd., Nanning, China; ^3^Guangxi Center for Animal Disease Control and Prevention, Nanning, China

**Keywords:** porcine respiratory coronavirus, porcine reproductive and respiratory syndrome virus, swine influenza virus, triplex crystal digital PCR, co-infection

## Abstract

Porcine respiratory coronavirus (PRCoV), porcine reproductive and respiratory syndrome virus (PRRSV), and swine influenza virus (SIV) are important pathogens of significant infectious diseases. They cause similar clinical respiratory symptoms, including fever, cough, runny nose, and respiratory distress, which makes these diseases difficult to distinguish from each other. In this study, three pairs of specific primers and TaqMan probes were designed for the conserved regions of the PRCoV S gene, PRRSV N gene, and SIV M gene, respectively. The annealing temperature, primer and probe concentrations, and reaction cycle were optimized, and a triplex crystal digital PCR (cdPCR) assay was established for the detection of PRCoV, PRRSV, and SIV. According to the test results, the assay was capable of specifically detecting PRCoV, PRRSV, and SIV, and there was no cross-reaction with other control swine viruses. Based on the Poisson distribution analysis, the limits of detection (LODs) for PRCoV, PRRSV, and SIV were 6.00, 5.75 and 6.00 copies/reaction, respectively, and the sensitivity was 26 times higher than those of the corresponding multiplex RT-qPCR. The coefficients of variation (CVs) of the intra-assay and inter-assay ranged from 0.19 to 1.84%. The assay was used to test 1,657 clinical samples, and the positivity rates of PRCoV, PRRSV, and SIV were 1.15, 12.79, and 2.05%, respectively. It showed diagnostic sensitivity and specificity of 100 and 99.82% for PRCoV, 100 and 99.24% for PRRSV, and 100 and 99.69% for SIV, respectively. These results indicated that the triplex cdPCR assay has strong specificity, high sensitivity, and excellent repeatability, which provides a valuable tool for the detection and differentiation of PRCoV, PRRSV, and SIV.

## 1 Introduction

With the development of intensive industrial farms and increasement of feeding density of pig industry, the respiratory infectious diseases have becoming important diseases that seriously endanger pig herds. Of which, porcine respiratory coronavirus (PRCoV), porcine reproductive and respiratory syndrome virus (PRRSV), and swine influenza virus (SIV) are important porcine pathogens which induce similar clinical respiratory symptoms of fever, cough, runny nose, and respiratory distress (1, 2). In serious cases, they can lead to high rates of morbidity and mortality, which cause great economic losses to the pig industry (3, 4).

PRCoV belongs to *Alphacoronavirus* genus in the *coronaviridae* family, which is an enveloped, single-stranded, positive-sense RNA virus with a 28 kb genome size (3, 5). PRCoV is considered as a naturally occurring deletion mutant of transmissible gastroenteritis virus (TGEV), with 5′ region deletion (nucleotides 621–681) in the Spike gene (6). After infection, it can lead to mild bronchial interstitial pneumonia and neutrophil infiltration, resulting in respiratory diseases in infected pigs (5, 7). The typical clinical manifestations include dyspnea, wheezing, sneezing, coughing, fever, anorexia, and growth retardation (8, 9). At present, PRCoV distributes in many countries around the world (5, 8, 10, 11), including China (12, 13).

PRRSV is a single-stranded, positive-sense RNA virus that belongs to the *Arterivirus* genus in the *Arteriviridae* family, and has a genome size of approximately 15.5 kb (14). According to genetic and antigenic characteristics, PRRSV is divided into European species (PRRSV-1) and American species (PRRSV-2). PRRSV-1 is represented by the Lelystad strain and PRRSV-2 is represented by the VR-2332 strain, and they have only about 60% similarity at nucleotide (15, 16). PRRSV cause immunosuppression, heightening the vulnerability to secondary infections and resulting in increased mortality rates in infected pigs (17, 18). The clinical manifestations include miscarriages, premature deliveries, and stillbirths in sows, as well as respiratory distress, hyperthermia, and fatalities in pigs (13). PRRSV has posed huge economic losses to the pig industry worldwide (19, 20). PRRSV was first reported in 1996 in China, and now has become a significant disease affecting pig herds throughout the country (21, 22).

SIV belongs to *Influenza A virus* genus in the *Orthomyxoviridae* family, and is a single-stranded, negative-sense RNA virus with a 13.6 kb genome size (23). It is a respiratory pathogen that is prevalent globally, and causes acute, febrile, and contagious respiratory disease in swine, and exhibits a high incidence of morbidity and low mortality (24). SIV is divided into different subtypes based on antigenic differences in glycoprotein haemagglutinin (HA) and neuraminidase (NA), with 18 subtypes for HA and 11 subtypes for NA (24). Nowadays, SIV is prevalent worldwide, and the mainly prevalent SIV subtypes are H1N1, H1N2, and H3N2, all of which show high diversity (25, 26). SIV was first reported in China in 1918, and to date, SIV has been discovered around the country (27, 28). Furthermore, SIV has been found in humans, and the trans-species transmission and zoonotic potential make serious threat to animals and humans (27, 29).

At present, PRCoV, PRRSV, and SIV are prevalent in many countries around the world (5, 8, 10, 11, 19, 20, 25–28, 30). Even worse, co-infections of PRCoV, PRRSV, and SIV occur in some pig herds, which exacerbates the seriousness of clinical manifestations and pathological damage, and induces more seriously economic losses to pig herds (17, 31–33). PRRSV/PRCoV co-infection was associated with reduced weight, higher incidence of fever, and more serious pneumonia compared with single infection; the infection of PRRSV followed by infection with PRCoV increased the damage of lymph nodes and lungs, and prolonged the persistent period of PRRSV, suggesting the synergistic effect of PRRSV and PRCoV (17). Co-infection with PRCoV and SIV may aggravate clinical signs and lung lesions (31, 32). PRRSV/SIV co-infection cause shortness of breath, dyspnea, fever, and cough, which can lead to more severe illness and growth retardation (33). PRRSV and SIV were found in pig herds (34). The positive antibodies against PRCoV, PRRSV, and SIV were found in German wild boar populations (35), and the positive nucleic acids of PRCoV, PRRSV, and SIV were found in clinical tissue samples in domestic pig herds in China (12). Since PRCoV, PRRSV, and SIV cause similar clinical signs of respiratory disease, the co-infections of two or three of these pathogens increase the complexity and difficulty of accurate diagnosis of these diseases. Therefore, development and application of a rapid, sensitive, and accurate technique for the detection and differentiation of these pathogens is necessary and helpful for early detection of the pathogens, accurate diagnosis of the diseases, and taking timely disposal measures, which is great significance for the prevention and control of these diseases.

The detection of viral nucleic acids is one of the important methods for accurate diagnosis of animal diseases. Of which, conventional RT-PCR/PCR, real-time quantitative RT-PCR/PCR, and digital RT-PCR/PCR are important techniques commonly used in veterinary laboratories (36, 37). Digital PCR (dPCR) is a third-generation PCR detection technology that has recently emerged, and is characterized by the separation of the reaction mixture into thousands to millions of partitions, and then perform amplification of real-time or endpoint detection (38). The target genes are partitioned according to the Poisson distribution model. At the end of the reaction, the target genes can be accurately and absolutely quantified according to the ratio of positive partition to all partitions (39). dPCR has the advantages of the ability to absolute quantification independent of reference gene, standard curve, and Ct value; higher resilience to inhibitors; high sensitivity, specificity, precision, and efficacy (40, 41). At present, dPCR is divided into droplet digital PCR (ddPCR) and crystal digital PCR (cdPCR), and have been used widely and effectively for the detection and quantification of swine pathogens, showing high sensitivity and accuracy (42–44). As for PRCoV, PRRSV, and SIV, the dPCR assays for the detection of PRRSV (45), classical + highly pathogenic + NADC30-like PRRSV (46), and ASFV + CSFV + PRRSV (47) have been reported. However, no multiplex dPCR assay has been reported for the simultaneous detection and differentiation of these three pathogens. In this study, a triplex cdPCR was developed for the detection of PRCoV, PRRSV, and SIV.

## 2 Materials and methods

### 2.1 Reference strains

The following vaccine strains were purchased from Harbin Harvac Biotechnology Co. Ltd. (Harbin, China): PRRSV (R98, Ch-1R and HuN4-F112 strains), classical swine fever virus (CSFV, CVCC AV1412 strain), TGEV (H strain), porcine epidemic diarrhea virus (PEDV, CV777 strain), and porcine rotavirus (PoRV, G5 type-NX strain). The following vaccine strains were purchased from Huapai Biotechnology Co. Ltd. (Chengdu, China): SIV (TJ strain), foot-and-mouth disease virus (FMDV, O/Mya98/XJ/2010 strain), and porcine circovirus type 2 (PCV2, ZJ/C strain). The clinical positive samples of PRCoV, PRRSV, African swine fever virus (ASFV), and porcine deltacoronavirus (PDCoV) were provided by our laboratory. All vaccine strains and clinical positive samples were stored at −80°C until use.

### 2.2 Clinical specimens

From July 2023 to March 2024, a total of 1,657 clinical samples from 1,657 pigs (the samples from each pig included trachea, lungs, lymph nodes, tonsils, and spleen) were collected from different pig farms, harmless treatment plants and slaughterhouses in Guangxi Province, China. The tissue samples from each pig were homogenized, and considered as one sample when tested for viral nucleic acids. All samples were stored at −80°C before use.

### 2.3 Primers and TaqMan probes

The genome sequences of 17 strains of PRCoV, 41 strains of PRRSV (16 strains of PRRSV-1, and 25 strains of PRRSV-2), and 40 strains of SIV were downloaded from the NCBI GenBank (https://www.ncbi.nlm.nih.gov/ (accessed on 15 April 2022)), and multiple sequence alignment was compared. Three pairs of primers and TaqMan probes were specifically designed for the conserved regions of the PRCoV S gene, PRRSV N gene, and SIV M gene using Oligo 7.0 primer design software as previous report by Ma et al. (12). The viral strains used for sequence comparison and the locations of the designed primers and probes were described in the supplementary figure S1 of a previous report (12). The information on the specific primers and TaqMan probes is shown in Table 1.

**Table 1:** The designed primers and probes for triplex cdPCR.

Pathogen	Gene	Primer/Probe	Sequence (5′ → 3′)	Product (bp)
PRCoV	S	PRCoV-F	TGGTTGTAATGCCATTG	85
		PRCoV-R	GCCACATAACTAGCACA	
		PRCoV-P	VIC-AAGTTTCCTACTTCYGTAGTTTC-BHQ1	
PRRSV	N	PRRSV-F	CCTCGTGYTGGGYGGCA	213
		PRRSV-R	GCTTCTCMGGSTTTTTCTT	
		PRRSV-P	FAM-TGGCCAGCCAGTCAATCARCTGTG-BHQ1	
SIV	M	SIV-F	CAAGACCAATCYTGTCACCTCT	91
		SIV-R	CGTCTACGCTGCAGTCC	
		SIV-P	CY5-TTCACGCTCACCGTGCCCAGT-BHQ3	

The degenerated primers were designed as follows: *M* = C/A, *R* = A/G, *S* = C/G, and *Y* = T/C.

### 2.4 Extraction of nucleic acid

The tissue samples of trachea, lungs, lymph nodes, tonsils, and spleen (about 0.1 g each tissue) were put into 2.0 mL EP tubes, and 1.0 mL of PBS buffer (pH 7.2) was added. The tissues were homogenized using a Retsch MM400 tissue homogenizer (Haan, Germany) with 30 Hz, frozen and thawed three times, and centrifuged at 12,000 rpm g for 5 min at 4°C. Then, 200 μL of the supernatant/vaccine solution was extracted using nucleic acid extractor (TIANGEN, Beijing, China) and Viral DNA/RNA Extraction Kit (TIANGEN, Beijing, China), and stored at −80°C.

### 2.5 Construction of standard plasmid constructs

The synthesized viral RNAs of PRCoV, PRRSV, and SIV corresponding to the target gene fragments for amplification were provided by Dalian TaKaRa Co. Ltd. (TaKaRa, Dalian, China), which were used for evaluating the sensitivity and repeatability of the developed triplex cdPCR assay.

All standard plasmid constructs were generated referencing the procedure described by Ma et al. (12). PRCoV, PRRSV, and SIV nucleic acids were extracted from positive samples or vaccine solution, reverse transcribed into cDNA using the PrimeScript II 1st Strand cDNA Synthesis Kit (TaKaRa, Dalian, China) according to the manufacturer’s instructions. The target fragments were amplified using three pairs of specific primers in Table 1, using cDNA as templates. After amplification, the target fragments were subjected to agarose gel electrophoresis, purified using MiniBEST DNA Fragment Purification Kit Ver.4.0 (TaKaRa, Dalian, China), cloned into pMD18-T vector (TaKaRa, Dalian, China), and transformed into *E. coli* DH5α competent cells (TaKaRa, Dalian, China). The positive clones were selected, added into LB solution, and incubated at 37°C for 20–24 h. The plasmid constructs were extracted from cultured bacterial fluids using MiniBEST Plasmid Extraction Kit Ver.5.0 (TaKaRa, Dalian, China), and named p-PRCoV, p-PRRSV, and p-SIV, respectively. The UV absorbance at 260/280 nm were measured using a spectrophotometer (Thermo Fisher, Waltham, MA, USA), and the concentrations of three plasmid constructs were calculated according to the following equation.


$$\mathrm{Plasmids}\left(\mathrm{copies}/\mathrm{\mu L}\right)=\frac{6.02\times 10^{23}\times \mathrm{plasmid}\;\mathrm{concentration}\times 10^{-9}}{\mathrm{plasmid}\;\mathrm{length}\left(\mathrm{bp}\right)\times 660}$$

### 2.6 Determination of reaction conditions

The Naica® Sapphire Crystal system (Stilla Technologies, Villejuif, France) was used to determine the optimal reaction conditions for the triplex cdPCR, including primer concentration, probe concentration, and annealing temperature. The total volume of 25 μL of reaction system was as follows: 12.5 μL 2× PerfeCTa Multiplex qPCR Tough Mix (Quanta Biosciences, Gaithersburg, MD, USA), 2.5 μL Fluorescein Sodium Salt (1 μM) (Apexbio Biotechnology, Beijing, China), three pair of primers and TaqMan probes with different final concentrations, 2.5 μL of the mixture of three standard plasmid constructs (final concentration of each plasmid construct was 10^4^ copies/μL), and distilled water to final volume of 25 μL. The annealing temperature of the triplex cdPCR was optimized from 55°C to 60°C, and the concentrations of primers and probes were optimized from 0.1 μM to 0.9 μM. The triplex cdPCR amplification procedure was as follows: 45°C for 5 min, 95°C for 10 s; then, 45 cycles of 95°C for 5 s, and 59°C for 34 s. After reaction, the chips were moved into the Naica™ Prism3 system (Stilla Technologies, Villejuif, France) to obtain images of VIC, FAM, and CY5 detection channels. According to the obtained droplets, the optimal reaction conditions were determined.

### 2.7 Generation of standard curve

The three plasmid constructs of p-PRCoV, p-PRRSV, and p-SIV were mixed at a ratio of 1:1:1, and 10-fold serially diluted from 1 × 10^5^ to 1 × 10^1^ copies/μL (final reaction concentration from 1 × 10^4^ to 1 × 10^0^ copies/μL). The standard curves were generated by performing triplex cdPCR using the optimal reaction conditions.

### 2.8 Specificity analysis

The extracted nucleic acids (RNA or DNA) of PRCoV, PRRSV, SIV, PRV, TGEV, PEDV, PoRV, ASFV, FMDV, PCV2, PDCoV, and CSFV were used as templates to analyze the specificity of the triplex cdPCR. The mixture of three plasmid constructs, the clinical negative sample, and distilled water were used as controls.

### 2.9 Sensitivity analysis

The synthesized viral RNA of PRCoV, PRRSV, and SIV were mixed at a ratio of 1:1:1, and 10-fold serially diluted from 1 × 10^5^ to 1 × 10^0^ copies/μL (final reaction concentration from 1 × 10^4^ to 1 × 10^−1^ copies/μL). The mixture was used as template to perform triplex cdPCR. The Poisson distribution analysis was used to evaluate the limits of detection (LODs) of the triplex cdPCR.

In addition, to further verify the results of the Poisson distribution analysis, the Probit regression analysis (https://www.ibm.com/cn-zh/spss) was also used to analyze the LODs of the assay. The mixture of PRCoV, PRRSV, and CSFV was 2-flod serially diluted as 31.23, 15.63, 7.81, 3.91, 1.96, 0.98, 0.49 copies/reaction, and used as templates. Each concentration was set for 20 repeats, and the times of positive amplification curve were counted. The Probit regression analysis was used to analyze the relationship between positive hit probability and detection concentration for evaluating the assay’s sensitivity.

### 2.10 Repeatability analysis

The synthesized viral RNA of PRCoV, PRRSV, and SIV were mixed at a ratio of 1:1:1, and diluted to 1 × 10^5^, 1 × 10^4^, and 1 × 10^3^ copies/μL (final reaction concentration of 1 × 10^4^, 1 × 10^3^, and 1 × 10^2^ copies/μL). The mixture was used as template to perform triplex cdPCR. The experiments were performed at three duplicates each time, and repeated at three weeks to assess the intra- and inter-assay variation of the triplex cdPCR.

### 2.11 Evaluation of the clinical samples

A total of 1,657 clinical samples collected between July 2023 and March 2024 in Guangxi Province of China were analyzed using the established triplex cdPCR. Meanwhile, these samples were also evaluated using the quadruplex RT-qPCR established by Ma et al. (12), which was used as a reference method for the simultaneous detection of PRCoV, PRRSV, and SIV. The diagnostic sensitivity and specificity of the developed triplex cdPCR were calculated. The consistency of the developed triplex cdPCR and the reference quadruplex RT-qPCR was assessed using SPSS 27.0 software.

## 3 Results

### 3.1 Standard plasmid constructs

After PCR amplification of the PRCoV S gene, PRRSV N gene, and SIV M gene using the specific primers in Table 1, the target fragments were purified, and connected to pMD18-T vector, then transformed into *E. coli* DH5α competent cells. The positive clones were screened and cultured, and the recombinant plasmid constructs were extracted and confirmed by sequence analysis. Three obtained plasmid constructs were named p-PRCoV, p-PRRSV, and p-SIV, respectively, and their concentrations were determined. The results showed that the initial concentrations of the plasmid constructs p-PRCoV, p-PRRSV, and p-SIV were 7.85 × 10^10^, 7.65 × 10^10^, and 6.35 × 10^10^ copies/μL, respectively. All standard plasmid constructs were diluted to 1.0 × 10^10^ copies/μL, and stored at −80°C until use.

### 3.2 Optimal parameters

**Figure 1:**
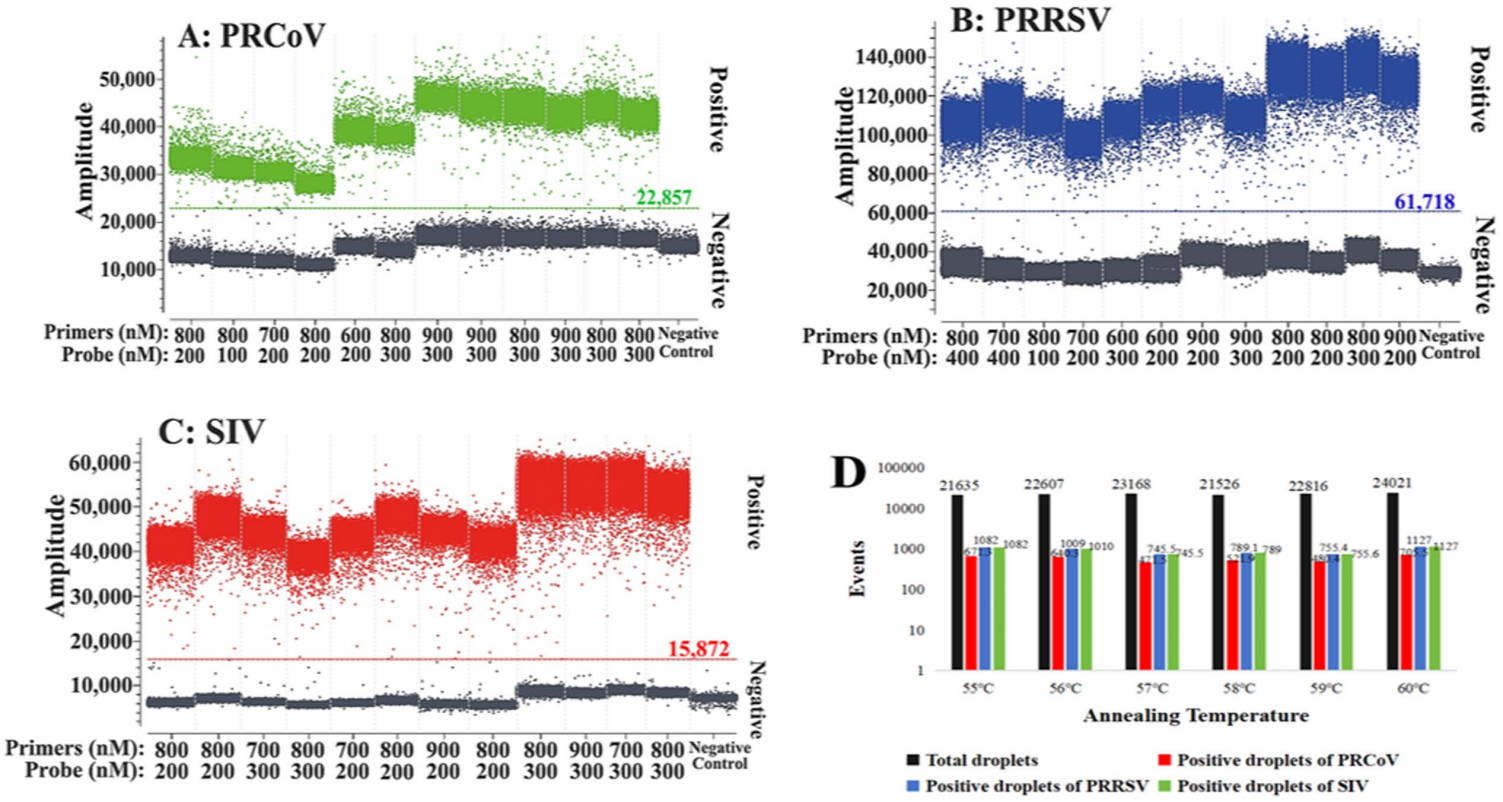
Optimization of the optimal reaction conditions for the triplex cdPCR. **(A–C)** Optimization of primer and probe concentration for PRCoV **(A)**, PRRSV **(B)**, and SIV **(C)**. **(D)** Optimization of annealing temperature.

To obtain the optimal reaction conditions, the annealing temperatures, primer and probe concentrations, and amplification cycles were adjusted to perform triplex cdPCR. The optimal reaction conditions were determined according to the following states of the reaction results: the largest number of total and positive droplets, the highest fluorescence signal values, the best droplet density, obvious distinction between positive and negative droplet clusters, and the least number of dispersed droplets between negative and positive droplets. Finally, the optimal reaction system of the triplex cdPCR is shown in Table 2. The amplification parameters were as follows: 45°C for 5 min, 95°C for 10 s, and then 45 cycles of 95°C for 5 s, and 60°C for 30 s. The triplex cdPCR achieved the best states of reaction results under the above optimal reaction conditions (Figure 1).

**Table 2:** The reaction system for the triplex cdPCR.

Reagent	Triplex cdPCR	
	Volume (μL)	Final concentration (nM)
PerfeCta multiplex qPCR ToughMix (2×)	12.5	1×
Fluorescein Sodium Salt (1 μM)	2.5	100
PRCoV-F (25 μM)	0.9	900
PRCoV-R (25 μM)	0.9	900
PRCoV-P (25 μM)	0.3	300
PRRSV-F (25 μM)	0.8	800
PRRSV-R (25 μM)	0.8	800
PRRSV-P (25 μM)	0.2	200
SIV-F (25 μM)	0.9	900
SIV-R (25 μM)	0.9	900
SIV-P (25 μM)	0.3	300
Total nucleic acid template	2.5	/
Nuclease-free distilled water	Up to 25	/

### 3.3 Standard curves

**Figure 2:**
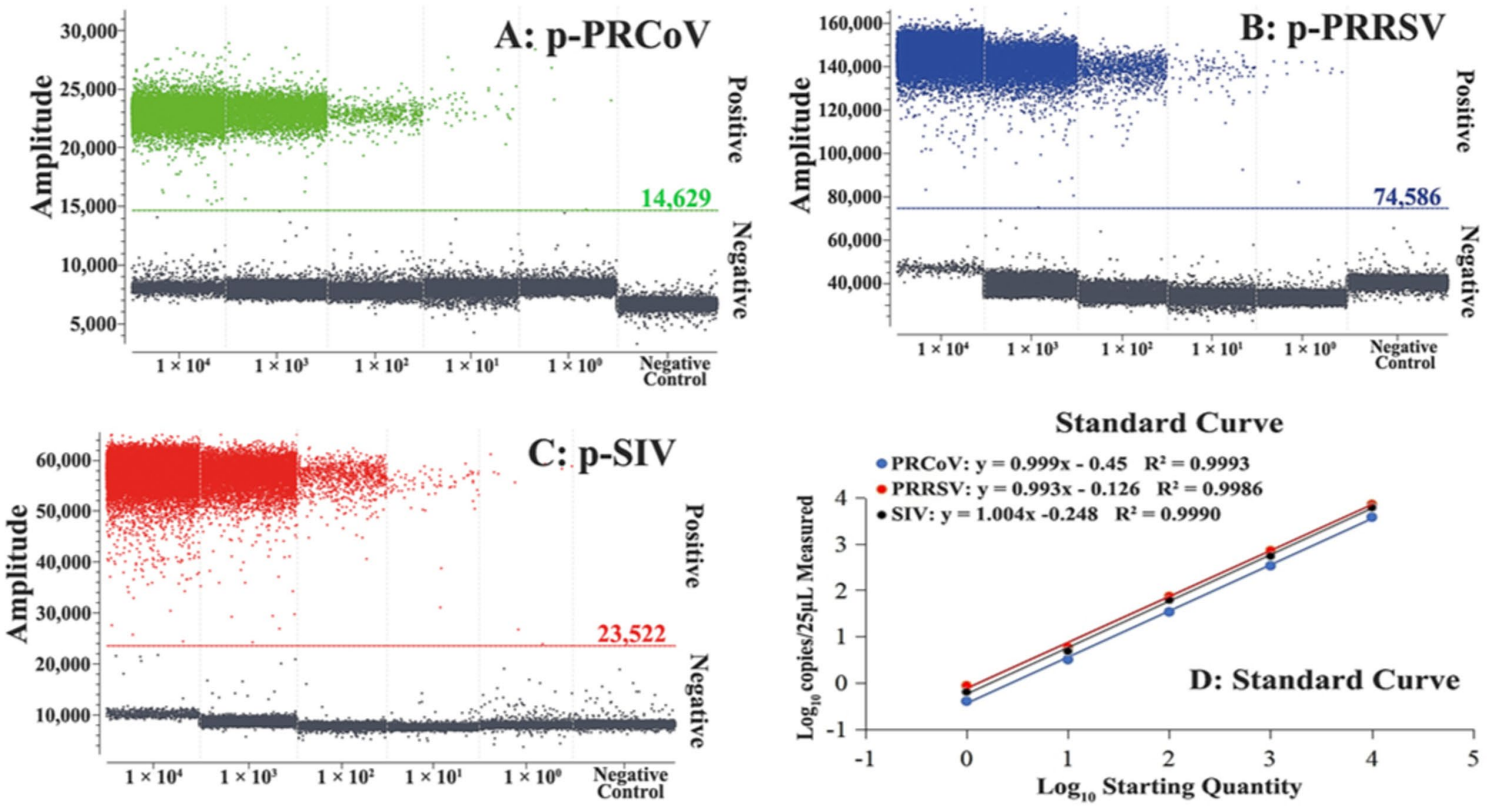
Generation of standard curves of the triplex cdPCR. The amplification plots of different final reaction concentrations (from 1 × 10^4^ to 1 × 10^0^ copies/μL) of plasmid constructs p-PRCoV **(A)**, p-PRRSV **(B)**, and p-SIV **(C)** and their standard curves **(D)** are shown.

The standard curves of the triplex cdPCR were generated using the mixture of three standard plasmid constructs from 1 × 10^5^ to 1 × 10^1^ copies/μL (final reaction concentration: from 1 × 10^4^ to 1 × 10^0^ copies/μL) as templates. The results showed that the slopes and the correlation coefficients (R^2^) of PRCoV, PRRSV, and SIV were 0.999 and 0.9993, 0.993 and 0.9986, and 1.004 and 0.9990, respectively (Figure 2), indicating excellent linear relationship between templates and positive droplets.

### 3.4 Specificity

**Figure 3:**
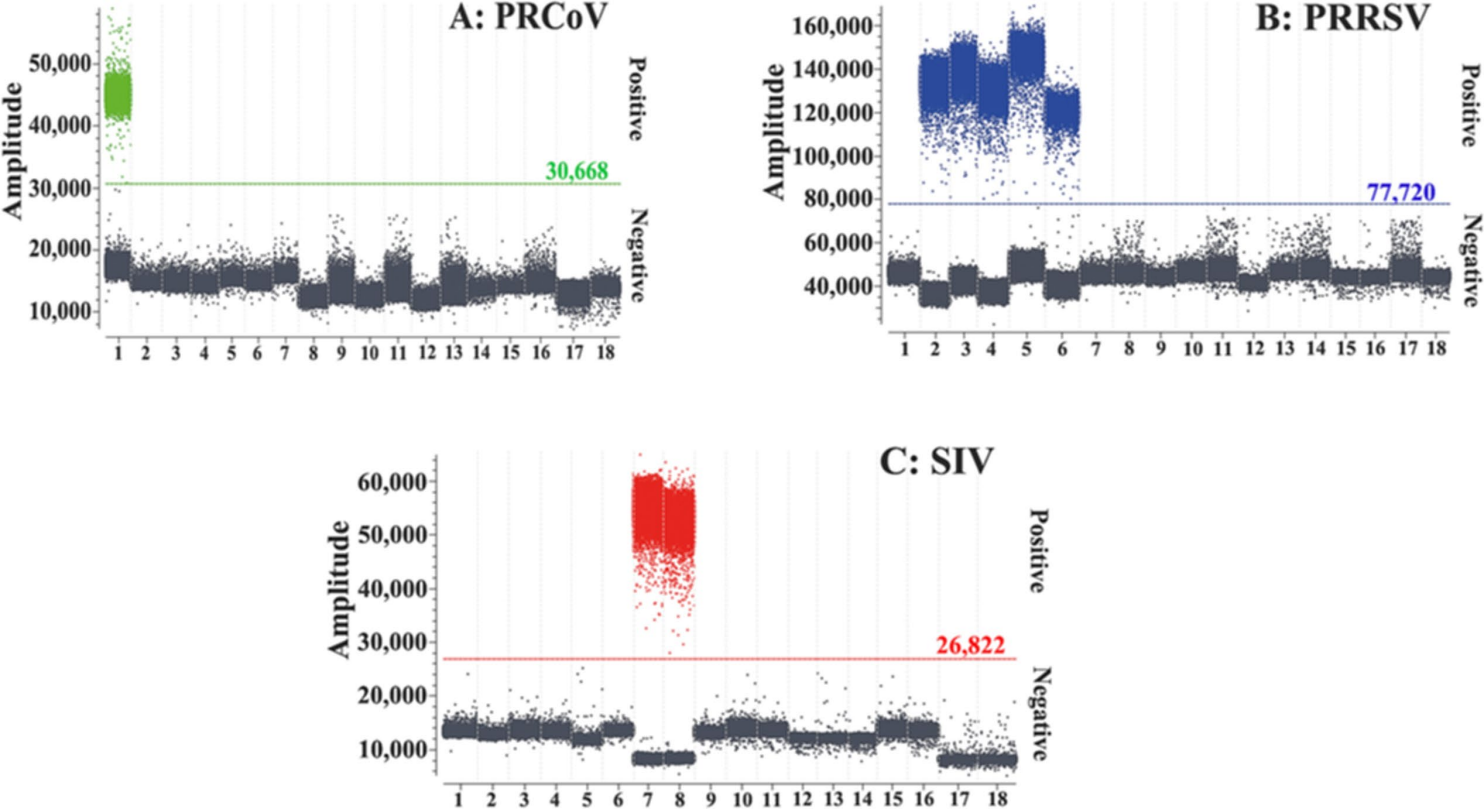
Specificity of the triplex cdPCR for PRCoV **(A)**, PRRSV **(B)**, and SIV **(C)**. 1: p-PRCoV; 2: p-PRRSV; 3: PRRSV; 4: PRRSV CH-1R strain; 5: PRRSV R98 strain; 6: PRRSV HuN4-F112 strain; 7: p-SIV; 8: SIV TJ strain; 9: TGEV H strain; 10: PEDV CV777 strain; 11: PoRV G5 type-NX strain; 12: FMDV O/Mya98/XJ/2010 strain; 13: PCV2 ZJ/C strain; 14: CSFV CVCC AV1412 strain; 15: ASFV; 16: PDCoV; 17: Clinical negative sample; 18: Nuclease-free distilled water.

The specificity of the triplex cdPCR was assessed using the extracted nucleic acids (RNA or DNA) of PRCoV, PRRSV, SIV, TGEV, PEDV, PoRV, ASFV, FMDV, PCV2, PDCoV, and CSFV as templates. The results showed that the triplex cdPCR could specifically detect PRCoV, PRRSV, and SIV, and there was no cross-reaction with other control swine viruses (Figure 3), indicating excellent specificity of the developed triplex cdPCR.

### 3.5 Sensitivity

**Figure 4:**
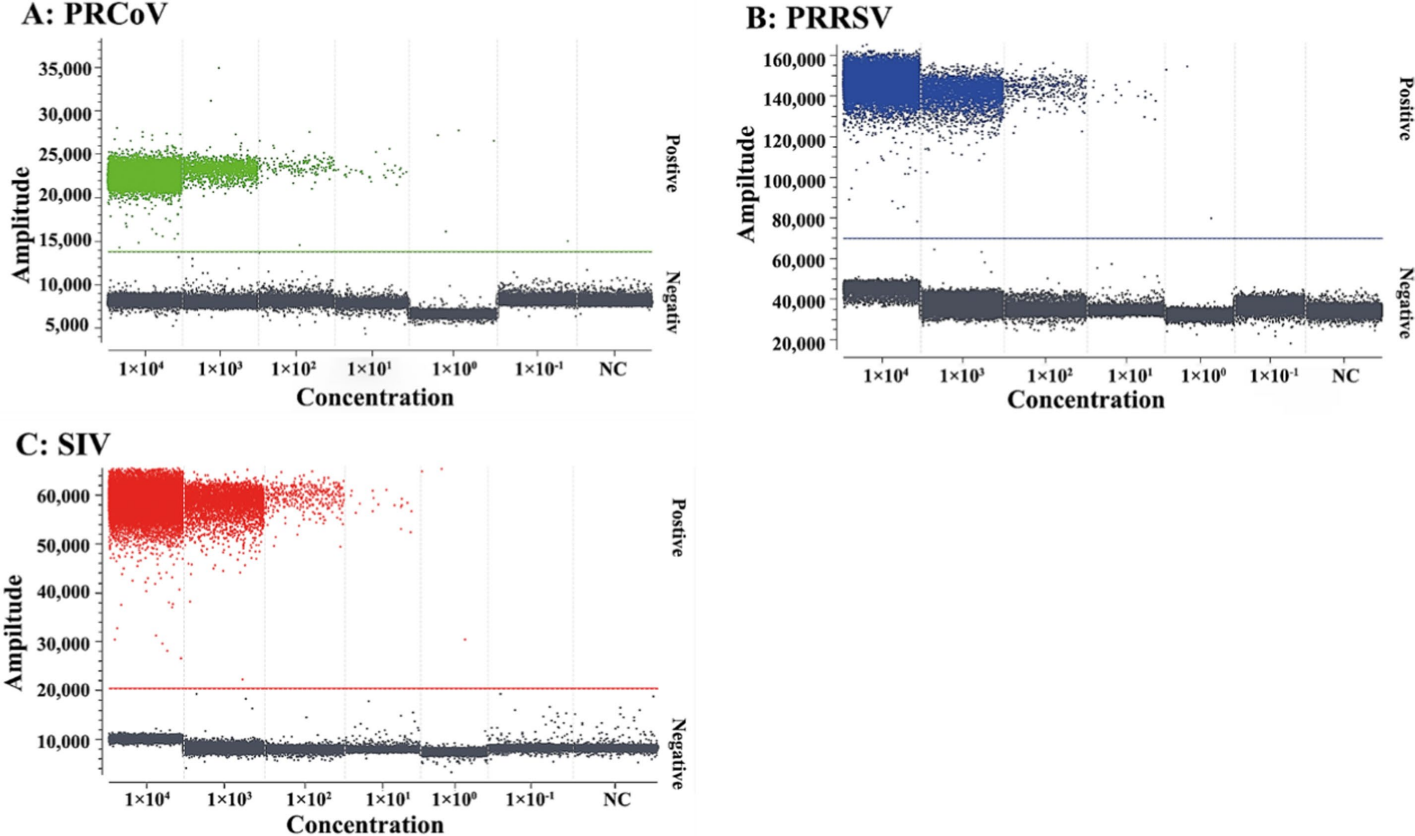
Sensitivity of the triplex cdPCR for PRCoV **(A)**, PRRSV **(B)**, and SIV **(C)**. The final reaction concentrations of the mixtures of synthesized viral RNA of PRCoV, PRRSV, and SIV ranged from 1 × 10^4^ to 1 × 10^−1^ copies/μL. NC, negative control.

The mixtures of three synthesized viral RNA of PRCoV, PRRSV, and SIV from 1 × 10^5^ to 1 × 10^0^ copies/μL (final reaction concentration: from 1 × 10^4^ to 1 × 10^−1^ copies/μL) were used as templates for evaluation of the sensitivity of the triplex cdPCR. The results basing on Poisson distribution analysis showed that the LODs of PRCoV, PRRSV, and SIV were 6.00, 5.75, and 6.00 copies/reaction, respectively (Figure 4).

**Figure 5:**
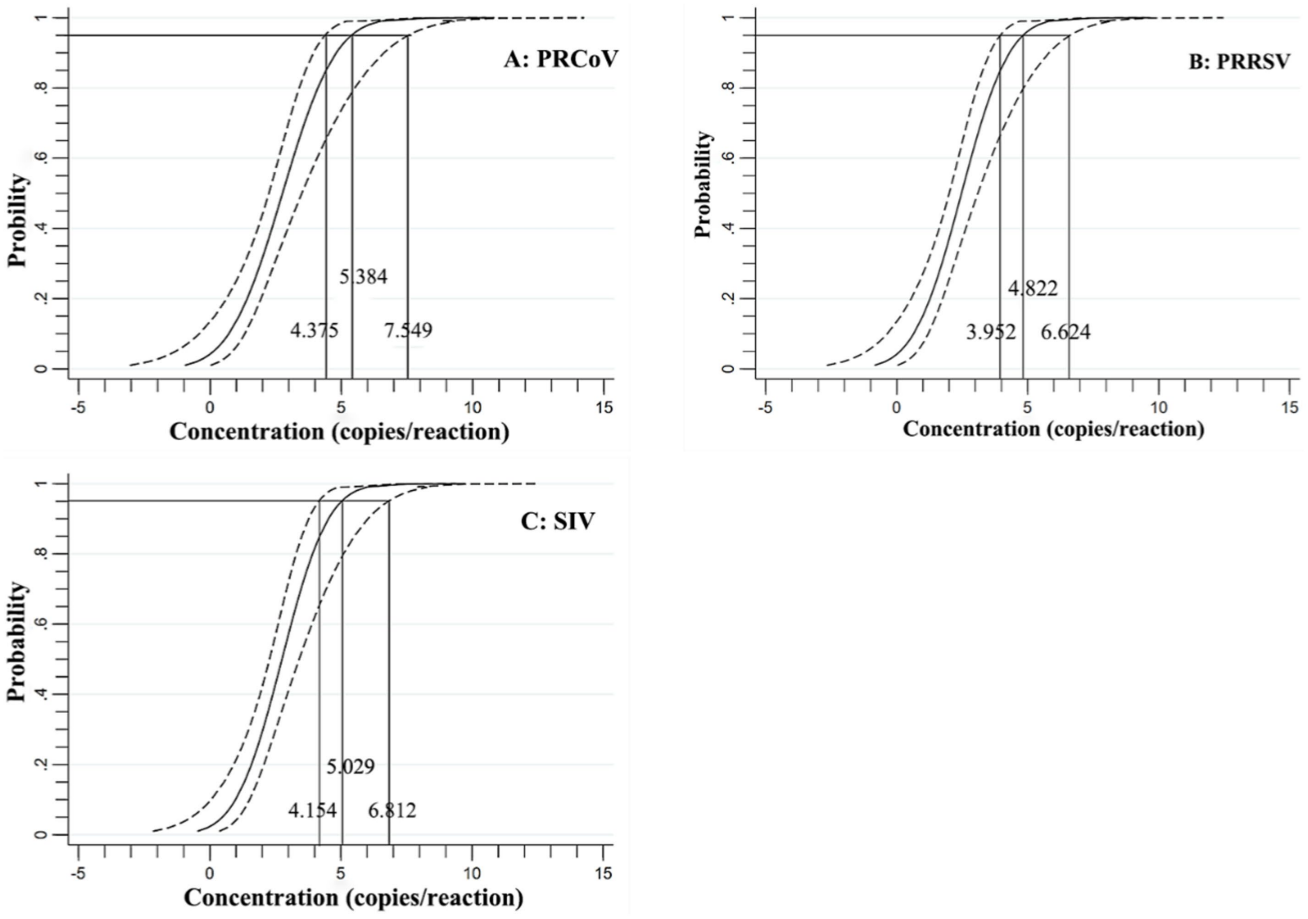
Specificity of the triplex cdPCR using Probit regression analysis for PRCoV **(A)**, PRRSV **(B)**, and SIV **(C)**.

In addition, the mixtures of three synthesized viral RNA of PRCoV, PRRSV, and SIV of 31.23, 15.63, 7.81, 3.91, 1.96, 0.98, 0.49 copies/reaction were used to performed triplex cdPCR, and the LODs were assessed using the Probit regression analysis. The number of positive droplets and hit rates were detected and recorded (Table 3). The LODs obtained for PRCoV, PRRSV, and SIV were 5.384, 4.822 and 5.029 copies/reaction, respectively (Figure 5).

**Table 3:** Sensitivity analysis of the triplex cdPCR with serial dilutions.

Concentration (copy/reaction)	Number of samples	PRCoV		PRRSV		SIV	
		Positive	Hit rate (%)	Positive	Hit rate (%)	Positive	Hit rate (%)
31.25	20	20	100	20	100	20	100
15.63	20	20	100	20	100	20	100
7.81	20	20	90	20	100	20	100
3.91	20	14	70	16	80	15	75
1.96	20	9	45	9	45	8	40
0.98	20	3	15	4	20	2	10
0.49	20	0	0	0	0	0	0

### 3.6 Repeatability

Repeatability of the triplex cdPCR was analyzed using the mixtures of three synthesized viral RNA of PRCoV, PRRSV, and SIV with final reaction concentration of 1 × 10^4^, 1 × 10^3^, and 1 × 10^2^ copies/μL as templates. The results showed that the coefficients of variation (CVs) were 0.44–1.46% for intra-assay and 0.41–1.67% for inter-assay (Table 4), indicating excellent repeatability.

**Table 4:** Repeatability analysis of the triplex cdPCR.

Viral RNA	Concentration (copies/μL)	Intra-assay (copies/reaction)			Inter-assay (copies/reaction)		
		$\bar{X}$	SD	CV (%)	$\bar{X}$	SD	CV (%)
PRCoV	1 × 10^4^	61500.00	682.83	1.11	62305.56	962.35	1.54
	1 × 10^3^	6126.667	39.65	0.65	6158.33	60.12	0.97
	1 × 10^2^	648.33	4.36	0.67	643.33	4.91	0.76
PRRSV	1 × 10^4^	73200.00	672.75	0.92	72602.78	454.01	0.63
	1 × 10^3^	6267.50	28.46	0.45	6021.11	24.74	0.41
	1 × 10^2^	711.67	3.12	0.44	690.56	6.41	0.93
SIV	1 × 10^4^	73041.67	496.46	0.68	72233.33	796.3	1.10
	1 × 10^3^	7421.25	65.60	0.88	7125.97	70.17	0.98
	1 × 10^2^	701.67	10.25	1.46	681.67	11.40	1.67

### 3.7 Test results of the clinical samples

**Figure 6:**
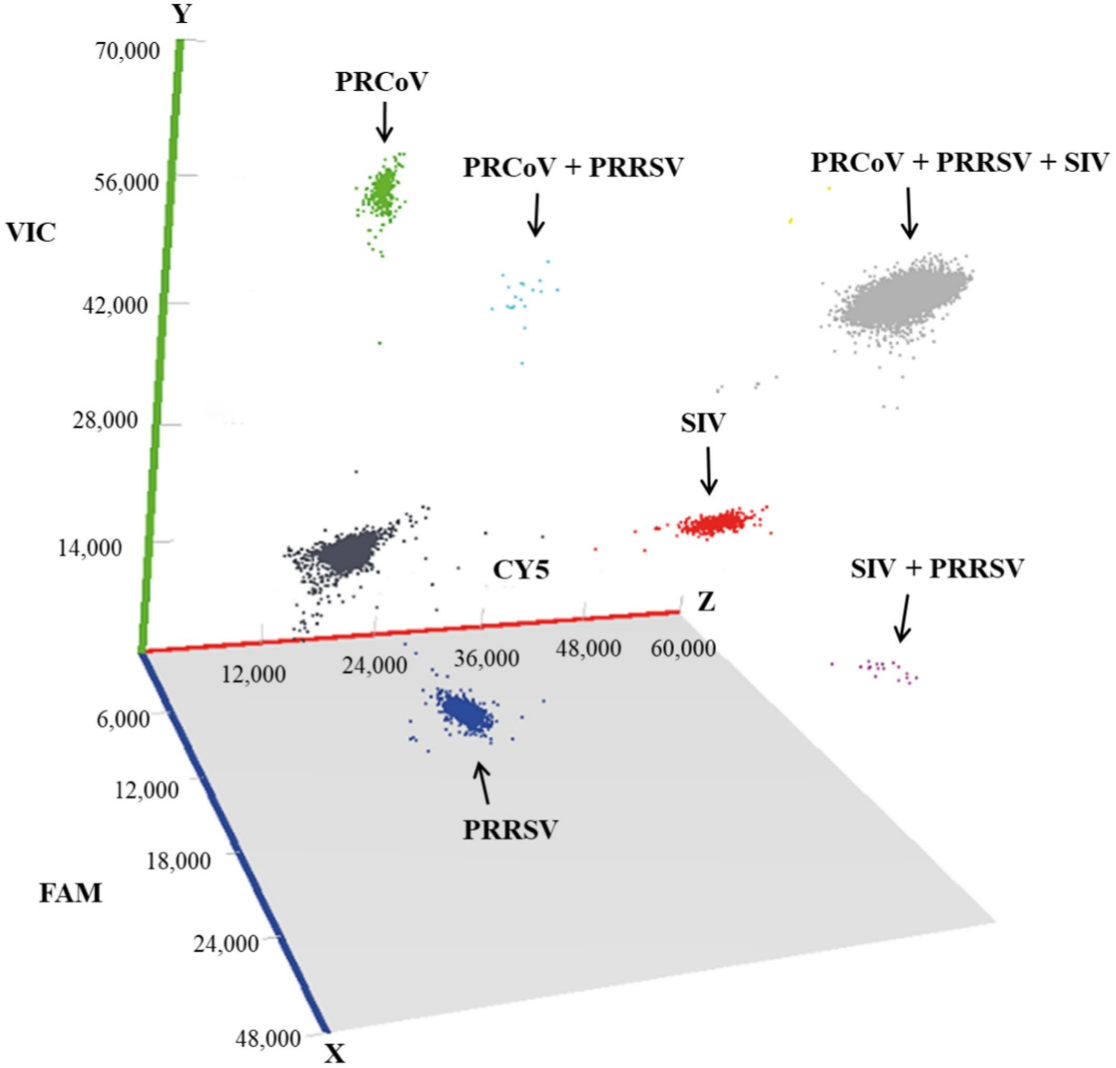
Test results of the clinical samples shown with 3D scatterplots. The fluorescence intensities were acquired in the Blue (x axis), Green (y axis) and Red (z axis) acquisition channels.

The 1,657 clinical samples collected from Guangxi Province in China from July 2023 to March 2024 were tested using the developed triplex cdPCR. The results showed that the positivity rates of PRCoV, PRRSV, and SIV were 1.15% (19/1,657), 12.79% (212/1,657), and 2.05% (34/1,657), respectively. The co-infection positivity rates of PRCoV + PRRSV + SIV, PRCoV + PRRSV, and PRRSV + SIV were 0.18% (3/1,657), 0.18% (3/1,657), and 0.12% (2/1,657), respectively. The positive samples were shown in a 3D dot-plots to display the data using three-dimensional scatterplots for direct visualization (Figure 6). The samples collected from pig farms, slaughterhouses, and harmless treatment plants showed positivity rates of 2.76, 0.56, and 3.06% for PRCoV, 8.76, 11.58, and 25.00% for PRRSV, and 3.23, 0.96, and 7.65% for SIV (Table 5).

**Table 5:** Test results of the clinical samples from different sources.

Source	Number	Number of positive sample (%)		
		PRCoV	PRRSV	SIV
Pig farm	217	6 (2.76%)	19 (8.76%)	7 (3.23%)
Slaughterhouse	1,244	7 (0.56%)	144 (11.58%)	12 (0.96%)
Harmless treatment plant	196	6 (3.06%)	49 (25.00%)	15 (7.65%)
Total	1,657	19 (1.15%)	212 (12.79%)	34 (2.05%)

The 1,657 samples were also tested using the reference multiplex RT-qPCR reported by Ma et al. (12). The results showed that the positivity rates of PRCoV, PRRSV, and SIV were 0.97% (16/1,657), 12.13% (201/1,657), and 1.75% (29/1,657), respectively. Compared with the reference multiplex RT-qPCR, the diagnostic sensitivity and specificity of the developed triplex cdPCR were 100 and 99.82% for PRCoV, 100 and 99.24% for PRRSV, and 100 and 99.69% for SIV, respectively (Table 6). The compliance rates of these two methods were higher than 99.34%, and the Kappa values of PRCoV, PRRSV, and SIV were 0.913, 0.970, and 0.919, respectively (Table 7).

**Table 6:** Diagnostic sensitivity and specificity of the triplex cdPCR.

The developed triplex cdPCR		The multiplex RT-qPCR		Total	Diagnostic sensitivity (95% CI)	Diagnostic specificity (95% CI)
		Positive	Negative			
PRCoV	Positive	16	3	19	100% (80.64–100%)	99.82% (99.46–99.94%)
	Negative	0	1,638	1,638		
	Total	16	1,641	1,657		
PRRSV	Positive	201	11	212	100% (98.12–100%)	99.24% (98.65–99.58%)
	Negative	0	1,445	1,445		
	Total	201	1,456	1,657		
PRCoV	Positive	29	5	34	100% (88.30–100%)	99.69% (99.28–99.87%)
	Negative	0	1,623	1,623		
	Total	29	1,628	1,657		

**Table 7:** Consistency analysis of the triplex cdPCR and the multiplex RT-qPCR.

Method	Positive sample		
	PRCoV (%)	PRRSV (%)	SIV (%)
The developed triplex cdPCR	19/1657 (1.15%)	212/1657 (12.79%)	34/1657 (2.05%)
The reference multiplex RT-qPCR	16/1657 (0.97%)	201/1657 (12.13%)	29/1657 (1.75%)
Positive agreement (95% CI)	100% (80.64–100%)	100% (98.12–100%)	100% (88.30–100%)
Negative agreement (95% CI)	99.82% (99.46–99.94%)	99.24% (98.65–99.58%)	99.69% (99.28–99.87%)
Overall agreement (95% CI)	99.82% (99.47–99.94%)	99.34% (98.82–99.63%)	99.70% (99.30–99.87%)
Kappa	0.913	0.970	0.919

## 4 Discussion

Digital PCR (dPCR) is a highly sensitive detection technology that can detect low concentration of target template (40, 41). The samples to be tested are diluted to form millions of droplet reaction chambers, where either one or no target template is distributed. Subsequently, PCR amplification is conducted and fluorescence signals are obtained. Finally, the absolute quantification of nucleic acid is achieved through Poisson distribution correction to obtain the copy number of nucleic acid (38, 39, 42–44). Compared with other assays, this method is able to accurately detect and quantify viral nucleic acids in samples without relying on standard curves, Ct values and amplification efficiencies. It is able to detect nucleic acids in samples with high sensitivity and accuracy, and exhibit low LOD (38–41). Therefore, dPCR has been gradually applicated in different veterinary laboratories for the detection of a variety of pathogens, which ensures the exact diagnosis of swine diseases. Unfortunately, the cdRT-PCR has the limitation of relatively high cost while compared with the RT-qPCR, but the development of multiplex cdPCR has decreased dramatically the average cost of each sample, so it has been applied in more and more laboratories now (47–49).

PRCoV, PRRSV, and SIV are important respiratory pathogens. Nowadays, these swine viruses circulate in many countries around the world (5, 8, 10, 11, 19, 20, 25–28, 30). Recently, these pathogens have been reported in many provinces in China, indicating that these pathogens are still circulating in many pig herds (12, 13, 21, 22, 27, 28, 30). They cause similar clinical respiratory symptoms of fever, cough, runny nose, and respiratory distress, and similar pathological damage of pneumonia. It is hard to differentially diagnose these diseases depending only on clinical signs and pathological changes. Therefore, it is necessary to establish specific, sensitive, and accurate method to detect and differentiate PRCoV, PRRSV, and SIV. To date, two dPCR assays for the detection of PRRSV (45, 46), and a triplex cdPCR assay for the detection of ASFV, CSFV, and PRRSV (47) have been reported. However, no multiplex cdPCR has been reported for the simultaneous detection and differentiation of PRCoV, PRRSV, and SIV.

In this study, three pairs of specific primers and TaqMan probes were designed for the conserved regions of PRCoV S gene, PRRSV N genes, and SIV M gene. After optimization of the optimal conditions of primer and probe concentrations, annealing temperatures, and reaction cycles, one triplex cdPCR for the simultaneous detection of PRCoV, PRRSV, and SIV was successfully developed. The assay showed strong specificity to detect PRCoV, PRRSV, and SIV, and high sensitivity to have very low LODs with about 5 copies/reaction for all three viruses, which is about 26 times higher sensitive than the corresponding multiplex RT-qPCR assay (about 130 copies/reaction) (12). In addition, basing on Poisson distribution analysis, the LODs of the individual cdPCR for PRCoV, PRRSV, and SIV in this study were 3.25, 3.25, and 3.25 copies/reaction, respectively, which were slightly higher sensitive than those of the corresponding triplex cdPCR. The assay had excellent repeatability with intra- and inter-assay CVs less than 2% (Table 4). The assay was used to test 1,657 clinical samples to evaluate its applicability, and showed diagnostic sensitivity and specificity higher than 99.24% for three viruses (Table 6). The assay had coincidence rate with the reference multiplex RT-qPCR higher than 99.34%, and the Kappa values were higher than 0.913 (Table 7). The results showed that the detection rates of PRCoV, PRRSV, and SIV were 1.15, 12.79, and 2.05% by the triplex cdPCR, and 0.97, 12.13, and 1.75% by the multiplex RT-qPCR. These results showed that the triplex cdPCR has a higher detection rate and it can be effectively applied in the clinical samples with low concentration of nucleic acid. These results indicated that a specific, sensitive, accurate, and reliable triplex cdPCR was established for the simultaneous and differential detection of PRCoV, PRRSV, and SIV.

The 1,657 clinical samples from Guangxi Province during 2023–2024 were tested using the developed triplex cdPCR, and the positivity rates of PRCoV, PRRSV, and SIV were 1.15, 12.79, and 2.05%, respectively. The results indicated that PRCoV, PRRSV, and SIV were still circulating in some pig herds in Guangxi Province, southern China. According to our previous surveillance, the positivity rates of PRRSV in Guangxi province from 2018 to 2023 ranged from 8.64 to 22.13% (unpublished data). The overall positivity rates of PRRSV in South China during 2017–2021 were 18.82% (1,279/6,795), ranging from 4.92 to 25% positivity rates (50). The suspected samples collected from Sichuan Province of China from 2021 to 2023 showed a prevalence rate of 39.74% (643/1,618) for PRRSV, with PRRSV-2 as dominant (95.65%, 615/643) and PRRSV-1 as minor (4.35%, 28/643) (51). The 7,518 samples collected from 100 intensive pig farms in 21 provinces in China during 2021–2022 showed a positivity rate of 32.1% (2,416/7,518) for PRRSV (52). At present, PRRSV-1 (European species) and PRRSV-2 (American species) are circulating in China (21, 51, 53), which increases the complexity and difficulty of prevention and control of PRRS. The surveillance of SIV in several regions of China during 2016–2021 found the highest positivity rate of Eurasian Avian-like (EA) H1N1 in pig farms, indicating that EA H1N1 has become an endemic subtype in the farm (54), and the EA H1N1 SIV was undergoing mutations and some of its genes were rearranged (55). In addition, SIV infection in humans has been reported (25, 29, 56, 57), which demonstrates the zoonotic potential of SIV strains and the importance of SIV surveillance. Compared with PRRSV and SIV, there has fewer reports on PRCoV at home and abroad until now. The 4,909 clinical specimens collected from Guangxi Province in China during 2022–2023 showed a positivity rate of 1.36% (67/4,909) for PRCoV (12). Two PRCV positive samples were found in the 7,645 samples from clinically healthy pigs at abattoirs in 13 provinces of China in 2017 (13). Further surveillance of PRCoV is necessary to take sufficient data on the endemic of PRCoV in China. Since the common prevalence of PRCoV, PRRSV, and SIV worldwide, the developed triplex cdPCR can be used as an important usefully tool to perform investigation and epidemiology of these pathogens, especially for the samples with very low viral loads.

Especially, the results showed the co-infection positivity rates of PRCoV + PRRSV + SIV, PRCoV + PRRSV, and PRRSV + SIV in these 1,657 samples were 0.18, 0.18, and 0.12%, respectively, which indicated that there were co-infections of two or three pathogens in pig herds. Co-infections of these viruses exacerbate the diseases (17, 31–35), which may be one of the reasons for the increased morbidity rate of pig herd. So, it is necessary to strengthen the surveillance of PRCoV, PRRSV, and SIV, to evaluate the epidemic situation of these pathogens, and to prevent and control these diseases effectively.

At present, PRCoV, PRRSV, and SIV are common prevalent in pig herds in China and other countries, and they induce similar clinical signs and pathological changes. Multiplex cdPCR assay is an important technical method for the detection of several pathogens in one reaction. The multiplex cdPCR assay established in this study provides a new technical tool for the clinical detection and differentiation of PRCoV, PRRSV, and SIV, which is beneficial to specially detect the existence of pathogens with very low concentration, and exactly diagnose the diseases. In addition, the specific primers and probes used in this study were designed for the conserved regions basing on the sequence alignment of the viral strains from different countries around the world, so the developed triplex cdPCR is suit for detection of the viral strains of PRCoV, PRRSV, and SIV from different countries.

## 5 Conclusion

A triplex cdPCR assay that can simultaneously detect and differentiate PRCoV, PRRSV, and SIV was successfully established for the first time in this study, which showed high sensitivity, strong specificity, and excellent repeatability. This assay can detect PRCoV, PRRSV, and SIV in the clinical samples with very low concentration in one reaction in a short time, and provide a powerful technical tool for rapid, and accurate detection of PRCoV, PRRSV, and SIV.

## Data Availability

The raw data supporting the conclusions of this article will be made available by the authors, without undue reservation.
